# Mesocellular Silica Foam as Immobilization Carrier for Production of Statin Precursors

**DOI:** 10.3390/ijms25041971

**Published:** 2024-02-06

**Authors:** Dino Skendrović, Mateja Primožič, Tonči Rezić, Ana Vrsalović Presečki

**Affiliations:** 1 Faculty of Chemical Engineering and Technology, University of Zagreb, HR-10000 Zagreb, Croatia; dskendrov@fkit.hr; 2 Faculty of Chemistry and Chemical Engineering, University of Maribor, 2000 Maribor, Slovenia; mateja.primozic@um.si; 3 Faculty of Food Technology and Biotechnology, University of Zagreb, HR-10000 Zagreb, Croatia; trezic@pbf.hr

**Keywords:** immobilization, aldolase, DERA, statins, mesoporous silica, pore size

## Abstract

The employment of 2-deoxyribose-5-phosphate aldolase (DERA) stands as a prevalent biocatalytic route for synthesizing statin side chains. The main problem with this pathway is the low stability of the enzyme. In this study, mesocellular silica foam (MCF) with different pore sizes was used as a carrier for the covalent immobilization of DERA. Different functionalizing and activating agents were tested and kinetic modeling was subsequently performed. The use of succinic anhydride as an activating agent resulted in an enzyme hyperactivation of approx. 140%, and the stability almost doubled compared to that of the free enzyme. It was also shown that the pore size of MCF has a decisive influence on the stability of the DERA enzyme.

## 1. Introduction

In the field of pharmaceutics, the synthesis of statins has attracted considerable attention due to their central role in the treatment of hypercholesterolemia and the prevention of cardiovascular disease [1,2,3,4,5]. Statins themselves are a class of cholesterol-lowering drugs that are essential in today’s medical treatment of cholesterol-related diseases [6]. Traditional chemical synthesis of statins often entails the use of hazardous reagents, leading to a multi-step, time-consuming process complicated by the presence of two chiral centers in statin molecules [7,8,9]. For the above reasons, there is a great need for a more environmentally friendly and economically viable method for the production of statins, which has led to the development of various biocatalytic synthetic routes that can be operational under mild reaction conditions and stereochemical purity [8,10,11]. There are several enzymes and methods for the biocatalytic production of statin intermediates in use today [2,9,12,13], but the most promising is the use of deoxyribose phosphate aldolase (DERA), which allows for the synthesis of both chiral centers in a one-step aldol addition reaction [14,15,16].

Despite the advantages of the DERA enzyme, there are still some major problems that preclude it from large-scale industrial application, namely, its high cost and sensitivity to high concentrations of reaction substrates and intermediates, which can lead to inhibition and inactivity [17,18,19]. Addressing this challenge involves various strategies, ranging from interventions at the enzyme level through techniques like directed evolution and site-directed mutagenesis [20,21,22] to enhancing enzyme stability in the reaction via diverse immobilization methods [23]. Immobilization offers a promising way to overcome DERA limitations. Immobilization of the enzyme on a solid support can improve both operational and storage stability, facilitates separation from the reaction mixture, and allows for reuse of the enzyme [24,25]. Among the various techniques available for the immobilization of enzymes on a solid support, covalent immobilization is considered one of the most effective, mainly because it is not prone to leaching of the enzyme due to the strength of the covalent bond, which is often a major problem with physical adsorption methods, making it suitable for industrial application [26]. When selecting materials to be used for the covalent immobilization, various requirements must be met, including thermal and mechanical stability of the carrier and a large surface area that can be functionalized with different reactive groups [23,26].

**Figure 1:**
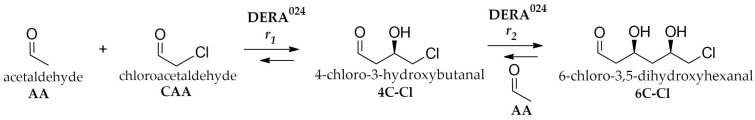
Reaction scheme of double aldol addition reaction for the synthesis of statin precursor.

Silica is an ideal candidate and is most commonly used in its mesoporous form, which is defined by a pore size of 2–50 nm [27]. It has the properties common for mesoporous materials, such as a large specific surface area (in the range of several hundred to one thousand m^2^/g), a large pore volume, the possibility to precisely define the pore size during synthesis, chemical stability, and the possibility of subsequent surface modification due to the high silanol content on its surface. In addition to the advantages and properties mentioned, it offers relatively undemanding synthesis routes, and it is possible to obtain significantly different structures with the same starting chemicals by changing the synthesis conditions [28,29,30]. Mesoporous silica can be broadly divided into two categories: Mobil Composition of Matter No. 41 (MCM-41) and Santa Barbara Amorphous-15 (SBA-15), depending on the surfactant and swelling agent used in the synthesis [28,31]. Mesocellular silica foam (MCF) is a type of SBA-15 obtained after its transformation (due to the addition of high amounts of swelling agent) from a highly ordered 2-D *p6mm* hexagonal structure to mesostructured cellular foam characterized by larger pore sizes connected with small windows and existing as a continuous 3-D structure [32,33]. Due to the larger pore size and pore volume present in MCF, there are indications that it may be more suitable for enzyme immobilization [34,35]. There is some research on the use of MCF as an immobilization carrier for DERA, such as two papers by Wang et al. [36,37], in which they successfully used DERA covalently immobilized to MCF for the synthesis of 2-deoxyribose 5-phosphate (DR5P) and achieved significantly higher activity and stability with this protocol compared to the free enzyme. However, there is a significant lack of research regarding the specific use of MCF-immobilized DERA in the double aldol addition of acetaldehyde and chloroacetaldehyde, which leads to the formation of statin precursors (Figure 1).

In this study, DERA was immobilized on MCF with different pore sizes. The focus was on the activity and stability of the immobilized enzyme during the reaction of the double aldol addition of acetaldehyde and chloroacetaldehyde (Figure 1). Since the functionalization and activating agents have a significant influence on the behavior of the enzyme during covalent immobilization, different combinations of each were tested. Upon identifying the most effective combination, kinetic parameters were estimated, and a mathematical model was developed and subsequently validated in a batch reactor. The synthesis of three distinct MCFs was conducted to investigate the influence of pore size on immobilization yield, retained activity, and stability of the enzyme. The performance of the DERA immobilized under optimal conditions and specific pore sizes was further assessed through multiple cycles in a batch reactor. Additionally, nitrogen adsorption/desorption analysis and scanning electron microscopy (SEM) were employed to verify the distinct structural and morphological properties of the synthesized MCFs.

## 2. Results

### 2.1. Characterization of Mesocellular Silica Foams

To get the three different types of MCFs used in this work, we varied only the Pluronic P123/1,2,4-trimethylbenzene (P123/TMB) ratio during MCF synthesis. SEM analysis was performed to evaluate the differences in particle morphology, whereas the adsorption/desorption technique was used to determine the pore diameter and specific surface area.

**Figure 2:**
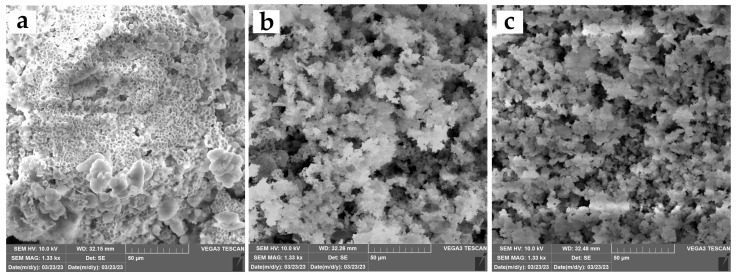
SEM images of MCFs synthesized by applying different P123/TMB ratios: (**a**) MCF-a; (**b**) MCF-b; (**c**) MCF-c. SEM analysis was conducted at 10 kV and 1.33 kx magnification.

The SEM analysis results are shown in Figure 2. The change in the P123/TMB ratio during MCF synthesis results in a distinctive variation in structure and morphology, leading from more ordered particles to significantly more disordered foam-like structure as the TMB concentration increased.

**Figure 3:**
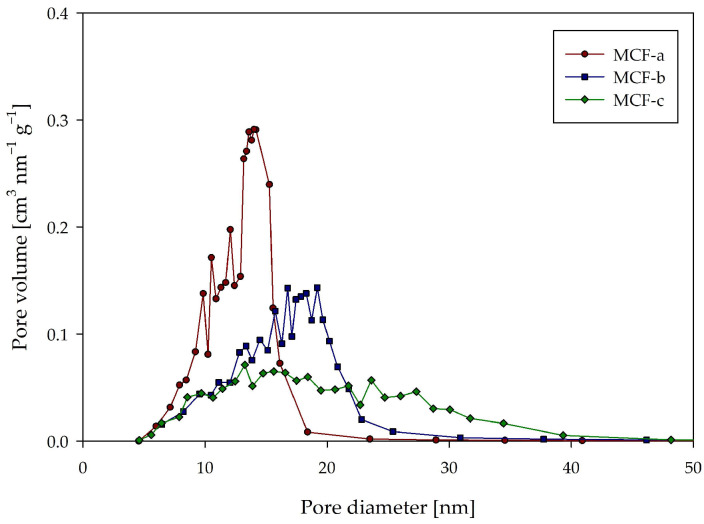
Pore size distribution from the BJH desorption isotherm.

The results of the nitrogen adsorption/desorption analysis (Table 1 and Figure 3) show that all three materials had high Brunauer–Emmett–Teller (BET) surface areas (298–209 m^2^/g) and high pore volumes (1.66–1.22 cm^3^/g) and that both parameters decreased with increasing pore diameter (Table 1).

Comparing the average pore diameters calculated using the Barrett–Joyner–Halenda (BJH) adsorption and desorption isotherms, there did not appear to be large differences (for MCF-a and MCF-c, the difference in average adsorption and desorption diameter was 4.7 nm and 4.3 nm, respectively). However, it is crucial to consider that all three materials exhibited a type IV isotherm and an H1 hysteresis loop [38,39] (Supplementary Material, Figure S1). This characteristic implies that the pore size distribution (PSD) from the BJH desorption isotherm held greater relevance and favorability than that from the BJH adsorption isotherm [40]. The PSD desorption isotherm for all three MCFs is shown in Figure 3. Here it can be clearly seen that, as expected, the range of available pore sizes increased in larger steps than with average pore diameters (Table 1). For MCF-a, the largest pore volumes fell in the diameter range of 12–16 nm and for MCF-b in the range of 18–22 nm, and MCF-c showed the widest distribution, with 15–35 nm. Considering these results and the fact that the hydrodynamic diameter of the DERA enzyme was around 9 nm [41], it was concluded that these three materials should be suitable as immobilization carriers for the DERA enzyme and that there were sufficient differences in the pore sizes to draw some relevant conclusions.

### 2.2. Influence of Functionalization and Activation Agents on Immobilization Parameters

Various combinations of functionalizing and activating agents were tested in order to chemically modify the surface of the MCF-a and thus create an optimal carrier for DERA immobilization. For a silica surface to be able to chemically bind with nucleophilic amino acid residues of the enzyme (in the case of DERA, the most common amino acid residue is lysine with ε-NH_2_ moiety), its silanol-rich surface must first be grafted with reactive electrophilic groups. This step consists of both functionalization, in which a type of organosilane is attached to the silanol groups on the silica to form siloxane bonds, and activation, in which an electrophilic group is covalently attached to these organosilanes [42]. In this study, 3-aminopropyltriethoxysilanes were used—both the methyl (APTMS) and the ethyl (APTES) variant, which are also the most commonly used organosilanes due to their cost-effectiveness and flexibility [26,43]. The activating agents tested with both organosilanes included benzoquinone, glutaraldehyde, and succinic anhydride—commonly employed for this type of immobilization and exhibiting promising outcomes in similar reactions [37,44,45,46].

**Figure 4:**
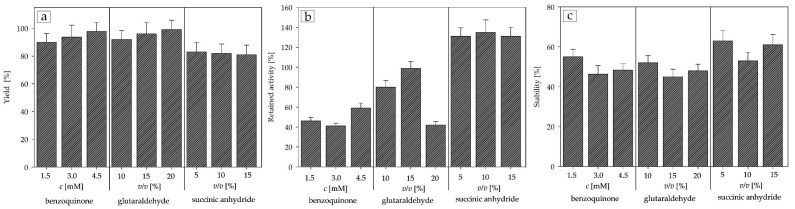
Influence of APTES-bound activation agents on (**a**) yield; (**b**) retained activity; (**c**) stability (0.1 M potassium phosphate buffer, pH 6, 25 °C).

Figure 4 shows the results of testing activating agents with APTES as a functionalization group. The immobilization yields for carriers activated with benzoquinone and glutaraldehyde were 85–95% of the bound enzyme, with 20% *v*/*v* glutaraldehyde giving the best result of 95%. A possible explanation for this is the crosslinking phenomenon that is common for glutaraldehyde-mediated immobilization [47] and its long spacer length [26]. Despite benzoquinone and glutaraldehyde yielding the highest immobilization yields, succinic anhydride outperformed in both retained activity and stability. The retained activity was notably higher with succinic anhydride activation, possibly due to hyperactivation inducing more positive conformational changes [48]. The stability of DERA immobilized on succinic anhydride-activated carriers reached approximately 60%, which is almost a twofold improvement over the stability of the free enzyme (37%).

**Figure 5:**
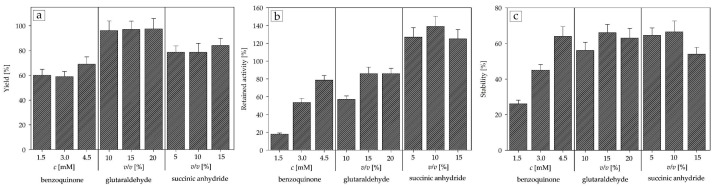
Influence of APTMS-bound activation agents on (**a**) yield; (**b**) retained activity; (**c**) stability (0.1 M potassium phosphate buffer, pH 6, 25 °C).

Figure 5 shows the results of the experiments with the APTMS functionalization group. These show a similar trend to the APTES functionalized carrier, with glutaraldehyde having the highest yield, but succinic anhydride still had a very clear advantage in the retained activity, with the stability remaining almost the same. Considering and comparing all the results obtained, MCF-APTMS-10% *v*/*v* succinic anhydride proved to be the optimal carrier, with 139% retained activity and 66% stability, and was employed for further testing and improvement.

### 2.3. Immobilized DERA^024^ Kinetics

The kinetic equations used and parameters obtained from the kinetic experiments (Figure S2) are shown in Table 2. The initial reaction method stated in Section 4.9. was used for all experiments. Comparing the results for the reaction of the first aldol addition, we can observe that the affinity (Michaelis constant *K_m_*) of the immobilized DERA^024^ was slightly lower towards both substrates, but the maximum reaction rate *V_m_* increased almost threefold, confirming the results of the previous section regarding the retained activity of succinic anhydride. As we hypothesized in our previous work with immobilized DERA^024^ [46], this was likely due to the reaction between succinic anhydride and the ε-NH_2_ moiety of DERA^024^ lysine, making the active sites of the enzyme more available to the substrates [49,50]. Regarding the reaction of the second aldol addition, there was a lower *V_m_* and slightly higher *Km* value for acetaldehyde, indicating lower affinity of the enzyme toward acetaldehyde, but both *K_i_* and *K_m_* values for intermediate 4-chloro-3-hydroxybutanal were noticeably better.

The mathematical model (Table 2, kinetic and batch reactor equations) was then validated in the batch reactor, as shown in Figure S3 (Supplementary Material). It is evident from the analysis that a solid convergence existed between the experimental observations and the model-generated data, a fact further substantiated by the statistical goodness-of-fit measures (σ = 7.29, *R*^2^ = 0.98), providing a foundation for further development and enhancement of this reaction in a batch reactor.

### 2.4. Influence of MCF Pore Size on Immobilization Parameters

**Figure 6:**
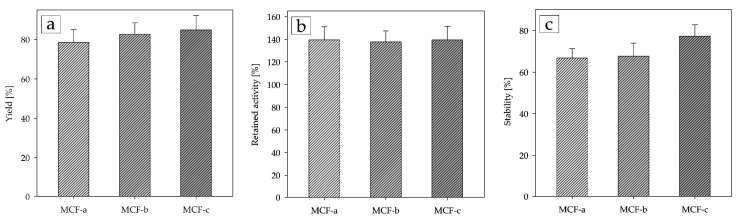
Comparison of (**a**) yield; (**b**) retained activity; (**c**) stability for the three types of MCF carriers (0.1 M potassium phosphate buffer, pH 6, 25 °C).

Having identified the optimal combination of functionalization and activating agent bound to the carrier MCF-a, the immobilization parameters were determined with MCF-b and MCF-c in order to investigate the influence of the pore size (Figure 6). When examining the yield results, there was a slight increase in loading capacity with increasing pore size from 78% for MCF-a to 85% for MCF-c, corresponding to an enzyme-loading capacity of 51 mg g^−1^ (10.34 µmol g^−1^) for MCF-c. There was almost no change in the retained activity, and all three carriers showed very similar results. The biggest difference was observed in stability, where there was only a 2% difference between MCF-a and MCF-b but a significant increase of 10% in MCF-c, resulting in a stability of about 77%. We believe that the main reason for this was that the larger pore size of MCF-c allowed the enzymes to penetrate deeper into the pore structure, protecting them from external physical and mechanical stresses [52] during the reaction and when washing the enzymes between reactions, which included the usage of high-speed shakers and centrifuges according to the protocol.

### 2.5. Operational Stability in Batch Reactor

**Figure 7:**
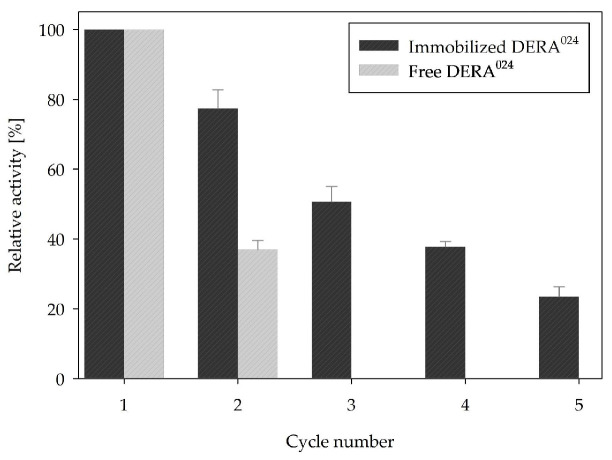
Comparison of relative activity of free and immobilized DERA^024^ in a sequential batch reactor (0.1 M potassium phosphate buffer, pH 6, 25 °C).

Operational stability was also tested in a batch reactor as a sequential process (Figure 7). As the MCF-c carrier in combination with APTMS-10% *v*/*v* succinic anhydride was shown to have the best stability, it was used for testing in the sequential batch process for five cycles of the aldol addition reaction (Figure 1). Whereas free DERA^024^ lost almost all its activity after the second cycle, immobilized DERA^024^ retained over 50% of its activity in the first three cycles and still retained 23% of its activity in the fifth cycle, indicating a noticeable improvement in DERA^024^ stability achieved by the optimized immobilization protocol.

## 3. Discussion

In this study, the first objective was to optimize the covalent immobilization method for DERA^024^ in the reaction of the double aldol addition of acetaldehyde to chloroacetaldehyde to produce statin side-chain precursors, using mesocellular silica foam as the carrier of choice. Covalent immobilization was selected based on the encouraging results of previous similar experiments with DERA [37,44,49] and our earlier findings [46]. Although the main issue with covalent immobilization is still the loss of enzyme activity [26,48,53,54], all tested concentrations of succinic anhydride seem to circumvent this problem and, at the same time, significantly improve enzyme activity and achieve hyperactivation (Figure 4 and Figure 5). Although there is no clear scientific explanation as to why this happens, there are a few hypotheses that seem to align with our particular case. Rodrigues et al. [48] postulate that the combination of short spacer arms of the activation agent, such as succinic anhydride [26], and a rigid support structure, as is the case with MCF, could lead to a strong stiffening of the enzyme structure, making it more resistant to various external stresses, while at the same time slightly altering the enzyme conformation, making more active sites available. Another possibility, briefly mentioned above in the Results section, is the fact that DERA is a class 1 aldolase, meaning that its reaction mechanism consists of the formation of a Schiff base intermediate between the donor substrate and the NH_2_ moieties of the amino acid residues of its active site [49], and succinic anhydride explicitly reacts with ε-NH_2_ lysine [50], which could potentially make the active sites more accessible to the substrates. Considering most of the available literature data on the immobilization of DERA [36,44,55,56,57,58], these results show a significant improvement in the retained activity with similar results in terms of stability and yield. However, even if our stability results are comparable to these previous data, it must be noted that there is a clear lack of literature on the immobilization of DERA in the reaction of double aldol addition, and here an almost twofold improvement over the free enzyme was obtained in all combinations tested.

The next step was to gain more insight into the reaction kinetics of the immobilized enzyme (Table 2). There are a few interesting observations to be made here. The most obvious is the much higher maximum reaction rate *V_m_*, which supports our conclusions regarding the hyperactivation of the enzyme. There is also the occurrence of parameter *n* (signifying number of molecules bound to the enzyme inhibition site) with both free and immobilized enzyme, which needed to be applied in the kinetic equations due to the occurrence of complete substrate inhibition (Table 2, Equation (1), Supplement Figure S2). This type of inhibition was first noted and explained in the findings of Bapiro et al. [59]. Even though this is a general limitation for carrying out the process with higher concentrations of chloroacetaldehyde, it can be easily solved by applying an appropriate reactor type [51]. The final notable observation is the apparently higher values of *K_m_* for the immobilized enzyme with both acetaldehyde and chloroacetaldehyde. As theorized by Rodrigues et al. [48] in their study, the influence of the immobilization process, which can alter the physicochemical properties of the enzyme environment, and the diffusion limitations imposed by porosity can significantly alter the distribution of substrates and products towards the enzyme. This can lead to a slower diffusion of the substrates towards the enzymes, which is reflected in the higher *K_m_* value. Although at first glance this appears to have a slightly negative effect, in our case and many others where substrate inhibition is present, it can be a net positive effect due to the minimization of contact between enzyme and inhibitor molecules.

After finding the ideal immobilization procedure, two other types of MCF were tested to evaluate the influence of porosity on the immobilization parameters (Figure 6). Although the retained activity remained almost the same for all three MCFs, we drew some interesting conclusions in terms of yield and stability. It appears that there was not much difference between the results for immobilization yield either, although MCF-c offered pores up to 35 nm in size compared to around 15 nm for MCF-a. This suggests that most enzymes crowded around the pore entrance, with protein–protein steric hindrances preventing other enzymes from penetrating deeper into the pores. Nevertheless, there was a 7% improvement from MCF-a to MCF-c, which, combined with the much lower surface area of MCF-c, could imply that the enzymes in MCF-c resided slightly deeper in the pore structures. This is also consistent with the significantly better stability of MCF-c, which could have been due to better protection against mechanical and physical stresses [52,53] but also due to different diffusion dynamics with the larger pore size and more disordered structure of MCF-c [48]. There was also an interesting review by Bayne et al. [35] that investigated the influence of pore size on immobilized enzymes by performing a rigorous statistical analysis of all published data on immobilization on mesoporous materials. They found that protein loadings increased with surface area up to 200 m^2^/g, after which they leveled out, and the same thing occurred with pore diameters above 10 nm, which is almost identical to our conclusions.

To discuss the results of the sequential batch reactor (Figure 7), a detailed comparison was made with our previous research on a similar topic [46]. There, immobilized DERA^024^ was also used in the double aldol addition reaction, but magnetic nanoparticles were employed as an immobilization carrier. Promising parallels can be observed regarding succinic anhydride as an activating agent in this particular reaction, as similar positive results were obtained in terms of immobilization parameters and enzyme kinetics. Although immobilized enzyme activity and stability in the batch reactor seemed to be lower, the immobilization yield of MCF was more than twice as high, which was probably due to its porosity. The reason for the lower stability in the batch reactor was probably the aggressive washing and centrifugation procedure between each cycle, which does not apply when using magnetic nanoparticles, as they can be separated by the magnetic field. Although this means that MCF may not be an ideal choice for this specific reaction in a batch reactor, it could be an ideal candidate for other reactor types due to its very high yield in combination with hyperactivation.

In conclusion, we believe that this study provides valuable new information both on the use of mesoporous silica as an immobilization carrier for DERA enzymes, with relevant insights into the various influences of porosity, and on the processes of statin side-chain synthesis using immobilized enzymes.

## 4. Materials and Methods

### 4.1. Chemicals

Acetaldehyde, 1,4-benzoquinone, Pluronic P123, 3-aminopropyltriethoxysilane (APTES) and (3-methylaminopropyl) trimethoxysilane (APTMS) were acquired from Acros Organics (Waltham, MA, USA). Potassium dihydrogen phosphate, dipotassium hydrogen phosphate, hydrochloric acid (HCl), and tetraethyl ortosilicate (TEOS) were obtained from Lach-Ner (Neratovice, Czech Republic). o-Benzylhydroxylamine hydrochloride was acquired from TCI (Oxford, UK). Bovine serum albumin (BSA), succinic anhydride, glutaraldehyde, and chloroacetaldehyde solution (50% (*w*/*w*)) were bought from Sigma-Aldrich (Darmstadt, Germany). Ethanol absolute was obtained from Scharlau (Barcelona, Spain). DERA^024^ from *Thermotoga maritima* was purchased from Prozomix (Haltwhistle, UK). Acetonitrile, trifluoracetic acid (TFA), and 1,2,4-Trimethylbenzene were acquired from Fisher Scientific (Loughborough, UK).

### 4.2. Synthesis of Mesocellular Silica Foams

The synthesis was performed according to the method proposed in the article by Chrzanowska et al. [33] In brief, 2 g of Pluronic P123 were dissolved in 90 mL of 1.6 M HCl solution and stirred at room temperature for 2 h. After 2 h, 2/5/10 g of TMB were added to the solution and stirred for another 2 h at 40 °C and 250 rpm. Then, 4/5/6 g of TEOS were added to the mixture. The solution was then stirred for 20 h at 40 °C and 180 rpm. Afterwards, the solution was placed in a Teflon-lined autoclave and kept in an oven heated to 120 °C for 24/96 h. The precipitate obtained was then filtered, rinsed with ultrapure water, and dried in air. The final step was the removal of the polymer template by calcification at 500 °C for 8 h. Synthesis conditions are provided in Table 3.

### 4.3. MCF Characterization

To assess the obtained morphology of the silica carriers, scanning electron microscopy (SEM) was performed on TESCAN VEGA3 (TESCAN, Brno, Czech Republic) operating at 10 kV. Prior to analysis, the samples spent 90 s in a magnetron ion-sputtering chamber to achieve the desired conductivity. To gain insight into the specific surface area, pore size, and pore volume of the silica carriers, N_2_ adsorption–desorption analysis was performed using the ASAP 2020MP (Micrometrics, Ottawa, Canada). Samples were degassed under vacuum at 70 °C until a stable pressure of 10 μm Hg was achieved. N_2_ was used as adsorption gas, and the analysis was carried out at −196 °C.

### 4.4. Functionalization and Activation of Immobilization Carriers

This process involved the functionalization of the silica surface via the introduction of amine groups, followed by the attachment of activating groups. To a solution of 1 g MCF dispersed in 50 mL of ethanol, 3 mL APTMS/APTES were added. The mixture was then stirred in an inert Ar atmosphere at 160 rpm and 30 °C for 24 h. The resulting MCF-NH_2_ precipitate was then washed with ethanol and dried at 60 °C for 24 h. Activation with glutaraldehyde consisted of stirring 25 mg of MCF-NH_2_ particles at 900 rpm for 15 h in a 1 mL potassium phosphate buffer (0.1 M, pH 6) solution with 10/15/20% GA. For p-benzoquinone activation, 25 mg of MCF-NH_2_ were stirred for 2 h at 900 rpm in a 1 mL potassium phosphate buffer (0.1 M, pH 6) solution containing 1.5/3/4.5 mM benzoquinone. Finally, succinic anhydride activation consisted of stirring 25 mg MCF-NH_2_ for 2 h in a 1 mL potassium phosphate buffer (0.1 M, pH 6) solution containing 5/10/15% succinic anhydride under an inert Ar atmosphere.

### 4.5. Immobilization of the Enzyme and Protein Concentration Measurements

Following each activation, enzyme immobilization was conducted by combining 10 mg of activated MCF with 1 mL of a 0.6 mg mL^−1^ DERA solution in potassium phosphate buffer (0.1 M, pH 6). This mixture was stirred for 2 h at 25 °C and 900 rpm. After this period, a supernatant sample was extracted to determine the quantity of unbound enzyme using the standard Bradford assay [60]. The immobilization yield was then determined using the following equation:

(7) $$Immobilization\;yield\;(\%)=\frac{c_{e}-c_{s}}{c_{e}}$$

### 4.6. Enzyme Assay

DERA activity was evaluated using the double aldol addition reaction shown in Figure 1. For each measurement, 100 μL of the free or immobilized enzyme solution was combined with 100 μL of aldehyde solution at 25 °C and 900 rpm. Samples were taken at periodic intervals within the first 20 min of the reaction and then analyzed by HPLC. The aldehyde solution contained 200 mM acetaldehyde and 100 mM chloroacetaldehyde in potassium phosphate buffer (0.1 M, pH 6). The determination of specific enzyme activity was carried out using Equation (8), where Δ*c*/Δ*t* describes the change in product concentration over time and *γ_e_* denotes the concentration of the enzyme. One mg/mL free enzyme activity correlates to 1.10 U/mL.

(8) $$S.\;A.=\frac{∆c}{∆t}\cdot \frac{1}{\gamma_{e}}$$

### 4.7. HPLC Analysis

To assess enzyme activity, substrate and product concentrations underwent analysis via HPLC by utilizing a Phenomenex Kinetex column (5 μm, C18, 100 Å, 250 × 4.6 mm), as described before [46].

### 4.8. Measurements of Stability and Retained Activity

Retained activity measurement was completed after each activation of MCF. Free and immobilized DERA activity was compared using Equation (9):

(9) $$Retained\;activity\;(\%)=\frac{Immobilized\;enzyme\;activity}{Free\;enzyme\;activity}$$

After finishing the first reaction with immobilized enzyme, MCF was washed three times with the potassium phosphate buffer (0.1 M, pH 6). The second reaction was carried out via the addition of 200 μL of aldehyde solution containing 100 mM acetaldehyde and 50 mM chloroacetaldehyde in the potassium phosphate buffer (0.1 M, pH 6). For measuring free enzyme activity in the second cycle, the enzyme was first washed and filtered using Amicon Ultra-0.5 Centrifugal Filter Units (MWCO 10 kDa), and the reaction was started via the addition of 200 μL of aldehyde solution containing 100 mM acetaldehyde and 50 mM chloroacetaldehyde in the potassium phosphate buffer (0.1 M, pH 6) to the filtered free enzyme residue. Both immobilized and free enzyme activity was then calculated using Equation (10):

(10) $$Stability(\%)=\frac{Enzyme\;activity\;in\;first\;cycle}{enzyme\;activity\;in\;second\;cycle}$$

### 4.9. Kinetic Analysis

Kinetic analysis was conducted for both steps of the reaction shown in Figure 1. Analysis was performed using the initial rate method, meaning that all samples were taken while the substrate conversion was below 10%, and all variables except the concentration of the monitored substrate were kept constant. The influence of each of the three substrates on specific enzyme activity was analyzed. Samples were taken within the first 5 min of each reaction, and HPLC was used to determine concentrations, as explained in Section 4.7. The concentrations used can be seen in Figure S2 (Supplementary Material).

### 4.10. Data Processing

Michaelis Menten kinetic parameters (*V_m_*, *K_m_*, *K_i_*, *n*) were approximated by employing the non-linear regression method available in the SCIENTIST 2.0 (MicroMath, Salt Lake City, UT, USA) software. To confirm model credibility, validation was conducted in the batch reactor. Coefficients of determination (R^2^) and standard deviations (*σ*) were used as measures of goodness of fit and were also calculated with the statistical functions available in SCIENTIST.

### 4.11. Batch Reactor Recycling Stability Measurements

Operational stability of the enzyme was tested in a batch reactor in the reaction of double aldol addition, employing the standard assay, as described above (Section 4.3). The reaction time was 15 min. The number of reaction cycles was five. Immobilized and free enzyme activity were set to 100%. After each reaction, the MCF carrier was washed three times with potassium phosphate buffer (0.1 M, pH 6) and then used again with the addition of fresh substrate. For free enzyme measurements, the mixture needed to be recovered by using Amicon Ultra-0.5 Centrifugal Filter Units (MWCO 10 kDa) after each reaction. It was then washed three times with potassium phosphate buffer (0.1 M, pH 6) and used in the next reaction cycle with the addition of fresh substrate. 

## Figures and Tables

**Table 1:** Physical properties of MCFs synthesized by applying different P123/TMB ratios.

Name	BET Surface Area (m^2^/g) *	Average Pore Diameter-BJH Adsorption (nm) *	Average Pore Diameter-BJH Desorption (nm) *	Pore Volume (cm^3^/g)
MCF-a	298.9 ± 4.6	19.8 ± 6.5	12.5 ± 3.3	1.66
MCF-b	225.9 ± 4.1	21.8 ± 7.2	15.2 ± 5.4	1.28
MCF-c	209.2 ± 4.2	24.5 ± 8.4	16.8 ± 4.1	1.22

* 95% confidence interval.

**Table 2:** Kinetic equations, batch reactor equations, and estimated kinetic parameters for both free and immobilized DERA^024^ in the reaction of double aldol addition.

Kinetic Equations	Parameter	Unit	Free DERA^024^ *	Immobilized DERA^024^
**First Aldol Addition**				
$r_{1}=\frac{{V_{m}}_{1}\cdot \gamma_{\mathrm{DERA}}\cdot c_{\mathrm{AA}}\cdot c_{\mathrm{CAA}}}{\left(K_{m\mathrm{AA}1}+c_{\mathrm{AA}}\right)\cdot \left({K_{m}}_{\mathrm{CAA}}+c_{\mathrm{CAA}}\cdot \left(1+{\left(\frac{c_{\mathrm{CAA}}}{{K_{iS}}_{\mathrm{CAA}}}\right)}^{n}\right)\right)}$ (1)	*V_m_*_1_	U mg^−1^	4.31 ± 0.63	12.50 ± 0.28
	*K_m_*_AA1_	mM	11.10 ± 2.54	30.61 ± 2.66
	*K_m_*_CAA_	mM	73.51 ± 9.11	215.04 ± 7.98
	*K_iS_*_CAA_	mM	260.96 ± 26.31	233.62 ± 3.11
	*n*	mM	20.00 ± 4.21	14.06 ± 3.02
**Second Aldol Addition**				
$r_{2}=\frac{{V_{m}}_{2}\cdot \gamma_{\mathrm{DERA}}\cdot c_{\mathrm{AA}}\cdot c_{4\mathrm{C-C1}}}{\left(K_{m\mathrm{AA}2}+c_{\mathrm{AA}}+\frac{{c_{\mathrm{AA}}}^{2}}{{K_{iS}}_{\mathrm{AA}}}\right)\cdot \left({K_{m}}_{4\mathrm{C-C1}}+c_{4\mathrm{C-C1}}+\frac{{c_{4\mathrm{C-C1}}}^{2}}{{K_{iS}}_{4\mathrm{C-C1}}}\right)}$ (2)	*V_m_*_2_	U mg^−1^	1.53 ± 0.26	1.11 ± 0.02
	*K_m_*_AA2_	mM	2.03 ± 0.28	14.97 ± 1.46
	*K_m_*_4C-Cl_	mM	86.26 ± 7.62	39.42 ± 11.97
	*K_is_*_AA_	mM	-	-
	*K_is_*_4C-Cl_	mM	72.44 ± 7.54	123.64 ± 10.56
**Batch Reactor Equations**				
$\frac{dc_{\mathrm{AA}}}{dt}=-r_{1}-r_{2}$ (3)	$\frac{dc_{\mathrm{CAA}}}{dt}=-r_{1}$ (4)	$\frac{dc_{4\mathrm{C-C1}}}{dt}=r_{1}$ (5)	$\frac{dc_{6\mathrm{C-C1}}}{dt}=r_{2}$ (6)	


* Data taken from Švarc et al. [51] and Skendrović et al. [46].

**Table 3:** MCF synthesis conditions.

Name	P123/TMB Mass Ratio (g/g)	P123/TEOS Mass Ratio (g/g)	Aging Time (h)
MCF-a	1:1	1:2	24
MCF-b	1:2.5	1:2.5	24
MCF-c	1:5	1:3	96

## Data Availability

Data will be made available on request.

## Supplementary Materials

The following supporting information can be downloaded at: https://www.mdpi.com/article/10.3390/ijms25041971/s1.
