# Supplementary material for: Mesocellular Silica Foam as Immobilization Carrier for Production of Statin Precursors
Source: Int J Mol Sci. 2024 Feb 6;25(4):1971. doi: 10.3390/ijms25041971 (PMC10887991; doi:10.3390/ijms25041971)
Supplement: Supplementary file 1 [file ijms-25-01971-s001.zip › ijms-2851590-supplementary.pdf]

Supplementary Material for

Mesocellular Silica Foam as Immobilization Carrier for Production of Statin Precursors

Dino Skendrović [1], Mateja Primožič [2], Tonči Rezić [3], Ana Vrsalović Presečki [1*]

1 University of Zagreb, Faculty of Chemical Engineering and Technology, 1000 Zagreb, Croatia; dskendrov@fkit.hr (D. S.); avrsalov@fkit.unizg.hr (A.V.P.)
2 Faculty of Chemistry and Chemical Engineering, University of Maribor, Smetanova Ulica 17, 2000 Maribor, Slovenia; mateja.primozic@um.si (M.P.)
3 University of Zagreb, Faculty of Food technology and Biotechnology, Pierottijeva 6, HR-10000 Zagreb, Croatia; trezic@pbf.hr (T.R.)
* Correspondence: avrsalov@fkit.unizg.hr; Tel.: +385 1 4597 157

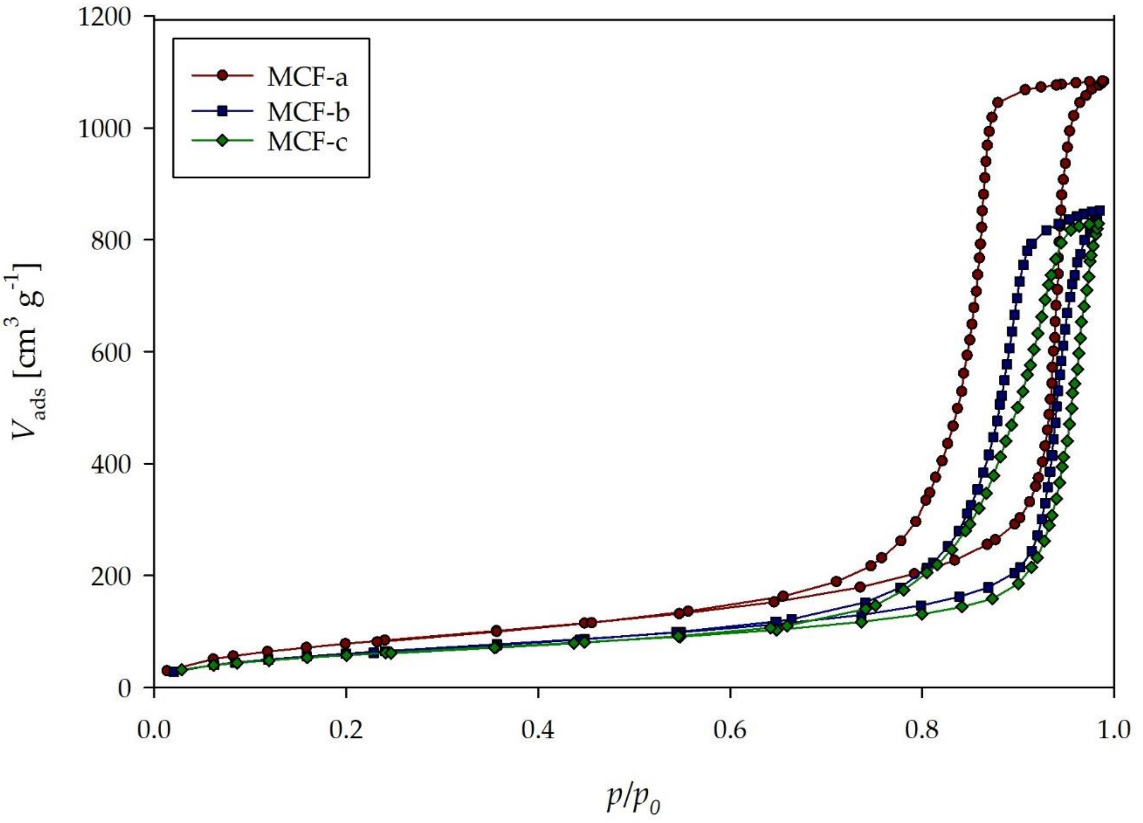

Figure S1. Nitrogen adsorption/desorption isotherms for the MCF carriers.

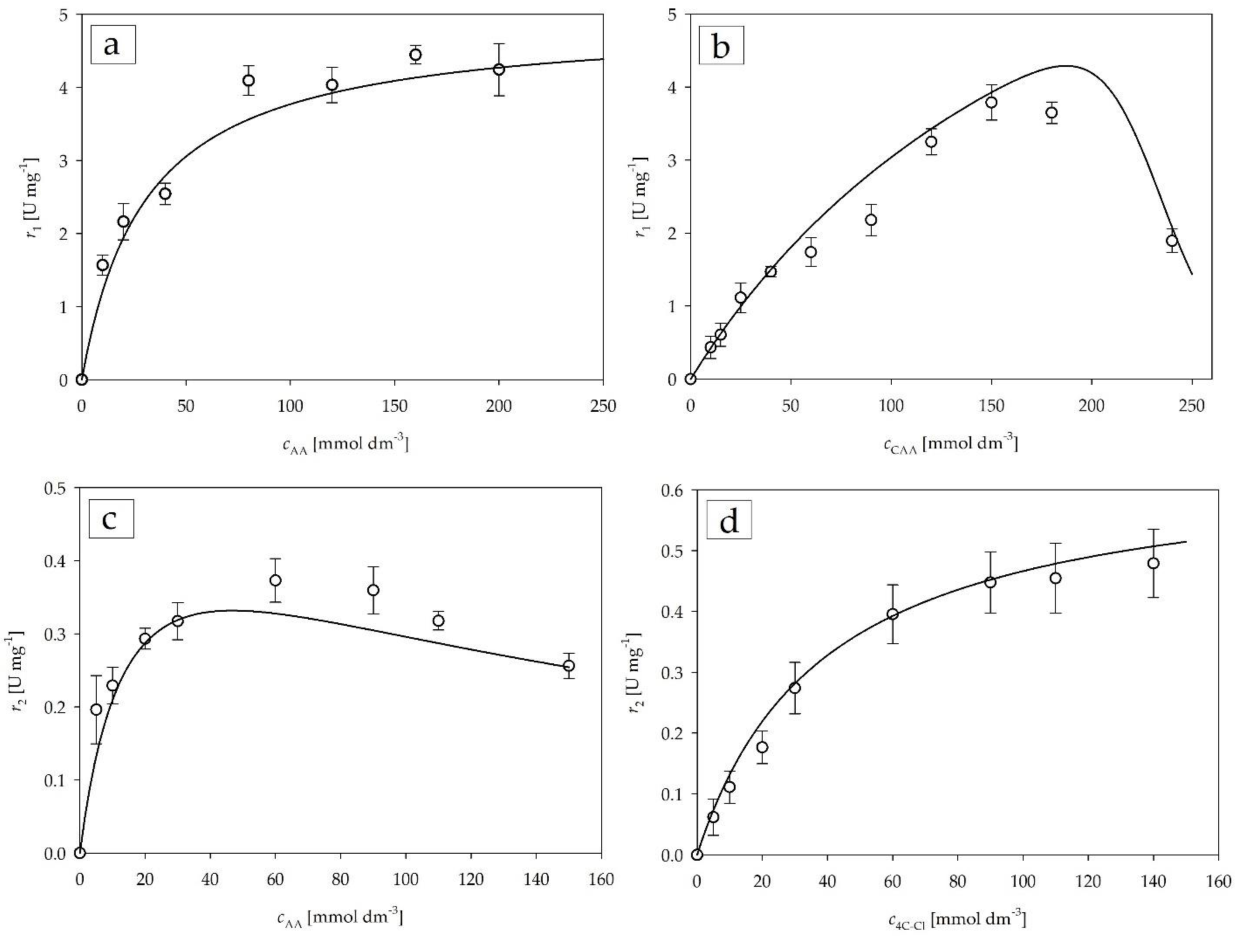

Figure S2. Kinetics of DERA immobilized on MCF-a/APTMS/10 % *v/v* succinic anhydride in the reaction of first (a, b) and second (c, d) aldol addition. The influence of: a -acetaldehyde ($c_{\text{chloroacetaldehyde}}$ = 100 mM), B - chloroacetaldehyde ($c_{\text{acetaldehyde}}$ = 100 mM), C - acetaldehyde ($c_{\text{4-chloro-3-hydroxybutanal}}$ = 5 mM) and D - 4-chloro-3-hydroxybutanal ($c_{\text{acetaldehyde}}$ = 40 mM) concentration on the initial reaction rate (0.1 M phosphate buffer pH 6, 25 °C, γ_{DERA} = 1 mg cm^{-3}).

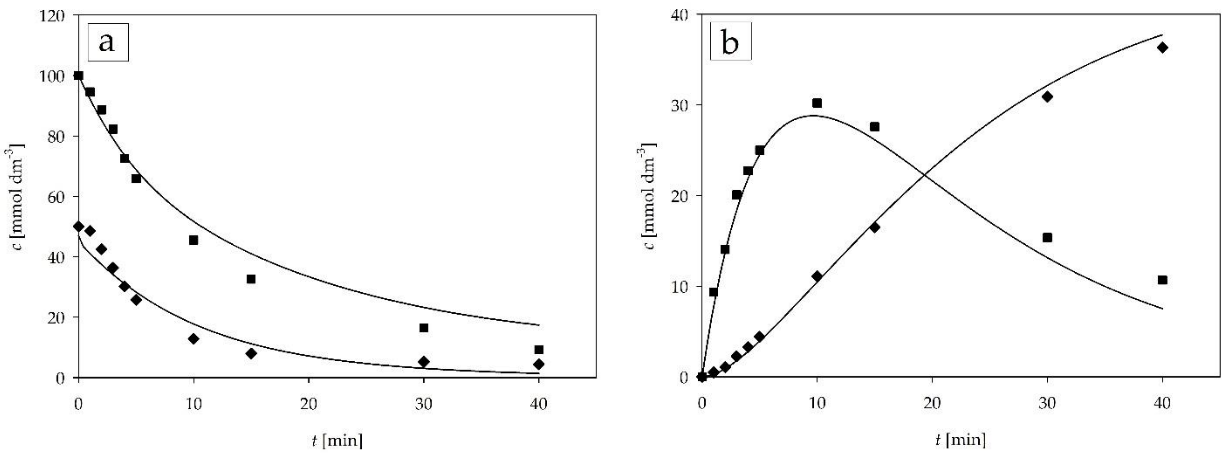

Figure S3. Mathematical model validation for double aldol addition reaction catalyzed by DERA[024] immobilized on MCF-a/APTMS/10 % *v/v* succinic anhydride in the batch reactor. Time change of (a) substrate (symbols: diamond – acetaldehyde, square – chloroacetaldehyde) and (b) product (symbols: square - 4-chloro-3-hydroxybutanal, diamond – lactol). ($c_{acetaldehyde}$ = 100 mM, $c_{chloroacetaldehyde}$ = 45 mM, 0.1 M phosphate buffer pH 6, 25 °C, γ_{DERA} = 2 mg cm^{-3}).
